# Malaria vaccines: identifying *Plasmodium falciparum* liver-stage targets

**DOI:** 10.3389/fmicb.2015.00965

**Published:** 2015-09-15

**Authors:** Rhea J. Longley, Adrian V. S. Hill, Alexandra J. Spencer

**Affiliations:** The Jenner Institute, Nuffield Department of Medicine, University of OxfordOxford, UK

**Keywords:** malaria, vaccine, liver-stage, T cells, immunity

## Abstract

The development of a highly efficacious and durable vaccine for malaria remains a top priority for global health researchers. Despite the huge rise in recognition of malaria as a global health problem and the concurrent rise in funding over the past 10–15 years, malaria continues to remain a widespread burden. The evidence of increasing resistance to anti-malarial drugs and insecticides is a growing concern. Hence, an efficacious and durable preventative vaccine for malaria is urgently needed. Vaccines are one of the most cost-effective tools and have successfully been used in the prevention and control of many diseases, however, the development of a vaccine for the *Plasmodium* parasite has proved difficult. Given the early success of whole sporozoite mosquito-bite delivered vaccination strategies, we know that a vaccine for malaria is an achievable goal, with sub-unit vaccines holding great promise as they are simple and cheap to both manufacture and deploy. However a major difficulty in development of sub-unit vaccines lies within choosing the appropriate antigenic target from the 5000 or so genes expressed by the parasite. Given the liver-stage of malaria represents a bottle-neck in the parasite’s life cycle, there is widespread agreement that a multi-component sub-unit malaria vaccine should preferably contain a liver-stage target. In this article we review progress in identifying and screening *Plasmodium falciparum* liver-stage targets for use in a malaria vaccine.

## Introduction

Malaria is a disease caused by the parasite *Plasmodium*, of which five species are known to infect humans, and is transmitted by the bite of female *Anopheles* mosquitoes. In 2013, approximately 584 000 people died from malaria (WHO, 2014), the majority due to *Plasmodium falciparum*. There has been substantial success in reducing mortality rates by approximately 50% over the past 15 years (WHO, 2014), but there is still a considerable burden of disease and some worrying trends in resistance to anti-malarial drugs. There are increasing reports of resistance to artemisinin in several countries of South East Asia (Ashley et al., 2014), and in addition reports of insecticide resistance to pyrethroids, the only insecticide class licensed for use in impregnated bed-nets, have started to emerge (Ranson et al., 2011). Together, these issues highlight the importance, and potential reliance the global community may have, on developing a highly efficacious and durable vaccine for malaria.

An effective whole parasite vaccination approach for malaria was developed in the early 1970s (Clyde et al., 1973; Rieckmann et al., 1974), building on previous work in animal models (Russell and Mohan, 1942; Nussenzweig et al., 1967). The parasite has a complex life cycle; within the human host, sporozoites injected by mosquitoes first travel to the liver. Here they develop for approximately 7 days, before entering into the bloodstream. The blood-stage is responsible for all symptoms associated with malaria, and gives rise to the sexual forms known as gametocytes. Researchers were able to demonstrate sterile protection – the complete absence of blood-stage parasites – after administration of irradiated sporozoites by mosquito bites. These irradiated sporozoites are able to invade the liver but development is arrested, providing a repertoire of antigens for the immune system to recognize without the host experiencing a blood-stage infection. Whilst this vaccination strategy was highly efficacious, it had numerous logistical issues, including the need to receive over 1000 bites from mosquitoes. Now, the biotechnology company Sanaria can cryopreserve sporozoites and vaccinate by needle-and-syringe, resulting in high levels of protection against subsequent challenge if 4–5 intravenous doses are administered (Seder et al., 2013), however, the durability of protection from this formulation has not yet been published. In addition, a number of manufacturing and logistical issues still remain, such as cost of manufacture, the route of administration, the need for storage in liquid nitrogen vapor phase and the requirement for large numbers of doses. An alternate, likely more practical, approach is sub-unit vaccination.

To-date, the most successful sub-unit vaccine is RTS,S Greenwood (2015), a particulate vaccine directed at the circumsporozoite protein (CSP). CSP is the major coat protein on the sporozoite surface (Yoshida et al., 1980; Nussenzweig and Nussenzweig, 1985) and is implicated in protection mediated by irradiated sporozoites (Gwadz et al., 1979; Nardin et al., 1982). The Phase III trial of RTS,S/AS01 conducted at eleven sites within seven African countries demonstrated 28% efficacy for 5–17 month-old children and 18% efficacy for 6–12 week-old infants with three doses, over the entire course of the study (∼3–4 years of follow-up; Greenwood, 2015). Our group has also had some success with a virally vectored sub-unit vaccine directed at the pre-erythrocytic antigen thrombospondin-related adhesion protein (TRAP) fused to a multi-epitope (ME) string (Gilbert et al., 1997; McConkey et al., 2003). When delivered in the vectors chimpanzee adenovirus 63 (ChAd63) and modified vaccinia virus Ankara (MVA), ME-TRAP provided 21% sterile protection in malaria-naïve adults, associated with CD8^+^ T cells inducing IFN-γ (Ewer et al., 2013). In the first field trial of this approach, significant efficacy (67%) in preventing PCR-detectable parasites was observed in Kenyan adults (Ogwang et al., 2015). Sub-unit vaccines with antigenic targets from the blood-stage of infection are also in clinical development (Drew and Beeson, 2015).

Given neither TRAP nor CSP alone (nor any blood-stage candidate) can match the protection induced by irradiated sporozoites, it is likely that a broad immune response to multiple target antigens will contribute to improved sub-unit vaccine efficacy. Only a minority of potential candidate antigens have been assessed as vaccine candidates, and CSP and TRAP may not be the best targets (Doolan et al., 2003; Kumar et al., 2006; Trieu et al., 2011). Furthermore, including multiple antigenic targets in one vaccine might overcome limitations in genetically restricted responses to certain epitopes (Doolan et al., 1996). The difficulty is choosing which antigenic targets from the 5000 or so genes expressed by the parasite (Gardner et al., 2002) should be incorporated. There is widespread agreement that a liver-stage target would be a desirable addition to a multi-component sub-unit malaria vaccine targeting another life-cycle stage, given that it represents a bottle-neck in the parasite’s life-cycle and could also be transmission blocking, by preventing development to the blood-stage and hence the formation of gametocytes.

In this article we therefore review what has been learnt so far in terms of identification and screening of novel liver-stage vaccine targets.

## Identifying Liver-Stage Targets

The discovery of the *Plasmodium* genome (Gardner et al., 2002) and proteome (Florens et al., 2002) has yielded a huge number of potential liver-stage targets, but has not necessarily provided information as to which should or could be included in a vaccine. In this section we will discuss a number of methods that could be used to prioritize antigens.

### Transcriptomic Profiling

Transcriptomic profiling of the *P. falciparum* liver-stage would uncover genes that are actively being expressed. This has, however, not been a simple task to undertake, even in murine models, given the high proportion of uninfected hepatocytes (Lau et al., 2001). Nevertheless, using techniques such as laser capture microdissection, axenically cultured exo-erythrocytic forms and fluorescent parasites, the *P. yoelii* transcriptome has been described, with around 1000 proteins likely expressed at the liver-stage (Wang et al., 2004; Sacci et al., 2005; Tarun et al., 2008). The *P. yoelii* liver-stage lasts for only 2 days, compared to 7 days for *P. falciparum*, and hence there are likely important differences in the transcriptomic profiles of these species. For example, the *P. falciparum* liver-stage specific protein liver-stage antigen 1 (LSA1) does not have a murine ortholog but is critical for late-liver-stage development (Mikolajczak et al., 2011).

Only one published study to our knowledge has attempted to define the *P. falciparum* liver-stage specific transcriptome. Siau et al. (2008) found 532 genes up-regulated following co-culture of *P. falciparum* sporozoites and primary human hepatocytes *in vitro* when compared to genes expressed in sporozoites alone. We recently demonstrated sterile protection following vaccination with one candidate identified in this study, liver-stage associated protein 2 (LSAP2; Longley et al., 2015b). This transcriptomic study was designed to identify genes important in the invasive period of the liver-stage, and hence they co-cultured the sporozoites and hepatocytes for only 1 h. Further research is now needed to determine the complete transcriptome, including late liver-stage development.

### Epitope Identification

To be immunogenic and targeted by T cells, the proteins need to be processed and epitopes presented on the hepatocyte cell surface in association with the major histocompatibility complex (MHC). Identifying which epitopes are presented at the liver-stage is hence also a rational method of choosing functionally important antigens. One method of epitope identification is prediction software (Trolle et al., 2015), used to predict the MHC binding capacity. This method has been applied to *P. falciparum* (Doolan et al., 2003) and reviewed in detail (Doolan, 2011) so will not be discussed further here, except to note that these predictions were made using the sporozoite proteome and that once the liver-stage specific *P. falciparum* proteome is known, they should be revisited. The risk with epitope prediction software is the rate of false positive identification (Zhong et al., 2003).

A direct method of epitope identification is the elution of peptides from *P. falciparum* infected hepatocytes. Generally, MHC-peptide complexes are isolated, the peptides eluted and separated by mass spectrometry, and subsequently sequenced (Hunt et al., 1992). This methodology has been used to identify epitopes for influenza (Testa et al., 2012), hepatitis C (Wolk et al., 2012), ovarian cancer (Ramakrishna et al., 2003), and tuberculosis (Flyer et al., 2002), but to our knowledge has not yet been used for *P. falciparum* due to the constraints on access to and infectability of hepatocytes.

Another method of epitope identification is a proprietary approach developed by Genocea Biosciences, Inc., known as ATLAS^TM^. They created a ‘high throughput, proteomic technology that facilitates unbiased and comprehensive identification of both CD4^+^ and CD8^+^ T cell antigens’ (Long et al., 2014). The rationale is to use whole proteins, rather than peptides, to stimulate immune cells from large numbers of individuals from two groups, those naturally protected from the pathogen and those susceptible. By developing a high-throughput technology able to screen large numbers of individuals they reduce the effect of HLA-restrictions. Furthermore, utilizing whole proteins, which are then processed by each volunteer’s own APCs, the resultant epitopes should be unbiased. This methodology is currently being explored for the identification of *P. falciparum* T cell targets.

### Source of Hepatocytes

The major limitations for both transcriptomic profiling and epitope identification are the source of infected hepatocytes and an absence of a perfect model of *P. falciparum* liver-stage infection. Whilst both hepatoma cell lines and primary human hepatocytes can be infected with *P. falciparum in vitro*, the infectivity rates are very low (∼0.1%; Mazier et al., 1985; Sattabongkot et al., 2006; March et al., 2013). In addition, *P. falciparum* sporozoites cannot naturally infect small rodents. Murine *Plasmodium* species are commonly used to study the liver-stage of infection in mice and *in vitro*, yet it is not clear how well these models reflect *P. falciparum* infections in humans. Whilst non-human primates can be infected with adapted human malaria parasites, they are not widely available and cost and ethical considerations of studies on higher order species are limiting factors.

An alternative model is the use of humanized mice; a number of laboratories now routinely infect various models of humanized mice with *P. falciparum* parasites (Morosan et al., 2006; Sacci et al., 2006; Vaughan et al., 2012b), after repopulation of 80–90% of the liver with human hepatocytes. The advantages over an *in vitro* model are that larger liver-stage parasites develop, up to 80 μm compared to 15–40 μm in culture (Mazier et al., 1985; Sattabongkot et al., 2006; Vaughan et al., 2012b). The larger size is more similar to results from studies of human infections (Shortt et al., 1951; Jeffery et al., 1952). Furthermore, liver-stage artifacts or abortive forms of parasites are often detected in cultures, yet this has not been observed in humanized mice. Whilst there are a number of disadvantages to this model (especially cost), it may be the most practical for obtaining large numbers of infected hepatocytes for antigen identification.

### Human Whole Sporozoite Immmunization for Antigen Identification

Once potential antigens or epitopes have been prioritized, cells from irradiated sporozoite immunization studies can be utilized to confirm that down-selected target antigens are immunogenic in vaccinated volunteers and to try and identify protective antigens. This is possible as protection in irradiated sporozoite vaccination models is dependent on T cells targeting the liver-stage (Schofield et al., 1987; Weiss et al., 1988; Hoffman et al., 1989; Seguin et al., 1994; Weiss and Jiang, 2012). Doolan et al. (2003) adopted this method to attempt to validate their antigens identified using epitope prediction software.

Alternatively, immunity can now be induced in humans with cryopreserved sporozoites (Sanaria^®^PfSPZ-CVac) or mosquito bite delivered sporozoites under drug cover (Roestenberg et al., 2011; Bijker et al., 2013). A major difficulty with this general approach is that of statistical power. With whole sporozoite strategies only tens of subjects are generally immunized and challenged and it is difficult to quantify degrees of protection precisely in each individual. Because there are hundreds of possible liver-stage antigens to be assayed, and most people respond weakly to most antigens, it is likely to be difficult to pinpoint the best individual protective antigen(s) standing out above the level of efficacy provided by the cumulative effects of large numbers of other antigens.

Unfortunately, this is also a limitation for the ATLAS^TM^ technology, as it relies on access to cells from humans whom are protected by whole pathogen exposure: for other pathogens they have used cells from exposed individuals (Long et al., 2014), whereas for malaria naturally exposed or challenged individuals generally have weak T cell responses (Offeddu et al., 2012; Sheehy et al., 2013). Hence, immunization models, such as described below, may be more useful.

## Screening Targets

Once candidate antigens have been identified, a functional assay to assess pre-clinical efficacy needs to be employed. The challenges are twofold: *P. falciparum* does not infect small animals, and there is no standardized *in vitro* assay to measure T cell killing of liver-stage parasites.

### Mouse Models

In the past, candidate antigens have been screened in mice using *P. berghei* or *P. yoelii* orthologs of the *P. falciparum* antigen of interest. It is still unclear how well these results translate to human trials, and importantly, not all *P. falciparum* antigens have murine orthologs. Alternate options are the use of transgenic parasites and humanized mice.

Transgenic parasites are a powerful tool that can enable functional screening of *P. falciparum* (or *P. vivax*) vaccine efficacy in mice (Persson et al., 2002; Mlambo et al., 2008; Cao et al., 2009; Espinosa et al., 2013; Porter et al., 2013; Bauza et al., 2014; Deal et al., 2014; Mizutani et al., 2014; Schwenk et al., 2014). Two methods are commonly used: (1) replacement of the endogenous *P. berghei* (or *P. yoelii*) gene with the *P. falciparum* ortholog under control of the relevant *P. berghei* promoter, or (2) addition of the *P. falciparum* copy of the gene inserted at a different and dispensable point in the genome (required when no murine ortholog exists). For example, the *230p* locus in both *P. berghei* and *P. yoelii* is considered “silent”, and replacement of this gene has no adverse effect on expression of other genes and no impact on parasite viability or behavior (Janse et al., 2006; van Dijk et al., 2010; Lin et al., 2011). We have recently used this method of screening to identify PfLSA1 and PfLSAP2 as potential candidates for a liver-stage malaria vaccine (Longley et al., 2015b): we were able to screen ten antigens for homologous efficacy in mice, and both PfLSA1 and PfLSAP2 outperformed CSP and TRAP in this model. We were also able to utilize the transgenic parasites to demonstrate that efficacy was primarily dependent on CD8^+^ T cells.

The main limitation of the addition rather than replacement technique is that the *P. falciparum* transgene is under control of a non-native promoter, potentially leading to differential patterns of gene expression. In addition, for both the replacement and addition strategies experiments are still undertaken in mice and the limited MHC system favors immunodominant responses, although this can be mitigated if outbred mice are studied. It is important to note that this is an artificial model system, and only once clinical trials of candidates selected in this manner have been undertaken successfully will we know its true value.

An alternative option is the use of humanized mice permissible to *P. falciparum* infection. As these mice are immunodeficient (Morosan et al., 2006; Sacci et al., 2006; Vaughan et al., 2012b) they would not be suitable for vaccination, but T cells from classical inbred mice could be adoptively transferred. To our knowledge this option has not yet been explored, but warrants some attention and preliminary experimentation to determine the feasibility.

### *In Vitro* Assays

*In vitro* assays could also be used for screening liver-stage vaccine candidates. *P. falciparum*-infected primary human hepatocytes and hepatoma cell lines have been used to measure antibody inhibition of invasion and growth (Mazier et al., 1986; Hollingdale et al., 1987, 1990; Mellouk et al., 1990; Fidock et al., 1997; Brahimi et al., 2001; House et al., 2009; Zou et al., 2013; Finney et al., 2014). To-date, only murine *Plasmodium* infected hepatocytes have been used to measure T cell-mediated protection (Hoffman et al., 1989; Weiss et al., 1990; Renia et al., 1991, 1993; Trimnell et al., 2009). T-cell assays require MHC effector-target cell matching, complicating the assay, and perhaps explaining in part why it has received little attention for many years. We have since revisited this assay, incorporating a number of technological advances such as fluorescent parasites to simplify the assay design and interpretation (Longley et al., 2015a). We utilized *P. berghei* TRAP as a model antigen and demonstrated TRAP-specific CD8^+^ T cell enriched splenocytes were able to inhibit *P. berghei* infected hepatoma cells in an effector-to-target ratio dependent manner.

However, the assessment of inhibition of *P. falciparum* infected human hepatocytes or hepatoma cell lines by human T cells is still an elusive goal. The difficulties are fourfold: (1) the rate of infectivity of *P. falciparum* sporozoites into human hepatocytes or hepatoma cells is inherently lower than that of murine *Plasmodium* species (Mazier et al., 1985; Sattabongkot et al., 2006); (2) *P. falciparum* sporozoites are more difficult to produce in the laboratory than murine species; (3) the lack of a *P. falciparum* parasite line with strong expression of a fluorescent or other visual marker throughout the entire lifecycle; and (4) the limited choice of human hepatoma cell lines with different HLA types (Karnasuta et al., 1995; Sattabongkot et al., 2006). Yet these barriers are constantly being reduced with improvements to liver-stage culture techniques (March et al., 2013; Zou et al., 2013; Ng et al., 2014) and the availability of cryopreserved *P. falciparum* parasites that could also reduce variability between assays (Sheehy et al., 2013). In addition, new *P. falciparum* parasites expressing fluorescent or luminescent markers are becoming available (Talman et al., 2010; Vaughan et al., 2012a), and flow cytometry detection of *P. falciparum* infected human hepatocytes and hepatoma cells has recently been demonstrated (Dumoulin et al., 2015). Finally the availability of cryopreserved primary human hepatocytes enriches the HLA-repertoire of target cells available (Li, 2014).

The remaining hurdle is the source of vaccine-induced human T cells. One option is through vaccination of human volunteers in clinical trials, however, this is not amenable to screening a large number of candidate antigens/vaccines. An alternate option is stimulation of naïve T cells *in vitro* (Gaucher et al., 2008), or alternatively (re-)stimulation of malaria-exposed T cells (for instance, sourced from whole sporozoite immunization studies, as described above).

The main advantage of using an *in vitro* system over *in vivo* murine models is the limited MHC repertoire of mice. Vaccination in mice often induces a response to only one immunodominant epitope; in humans, there are multiple epitopes that are often different between vaccinated individuals. However, as mentioned above, this can be overcome using outbred mice, although this still does not reflect the HLA-types of a human population. In turn, the *in vitro* model is still an artificial system, and likely does not recapitulate all immunological processes (such as signaling pathways) that would occur *in vivo.* Ultimately, screening liver-stage targets in both models (*in vivo* and *in vitro*) would be preferential.

### Immune Correlates of Protection

Ideally, these methods would not only provide a useful model for screening liver-stage vaccine candidates, but also be used to define correlates of protection. If any of the functional assays of immunity described above (*in vivo* or *in vitro*) could accurately predict which vaccines provided protection in CHMI trials, the assay could then be used as a surrogate for protection. This could potentially alleviate the need for very large and expensive CHMI trials at the initial stages of vaccine assessment and would be extremely beneficial for the development of second-generation malaria vaccines.

## Summary and Conclusion

In summary, in the current absence of a deployable and highly efficacious malaria vaccine, significant advances are enabling the identification of new *P. falciparum* liver-stage vaccine targets. Various antigen identification platforms are helping to reduce the number of potential candidates, and simultaneously, methods to screen such candidates are greatly improving. It will be of great interest to see whether any recently identified candidates, such as PfLSAP2, translate into protective efficacy in CHMI trials of non-immune adults. We believe these new identification and screening platforms will greatly enhance the development of second-generation malaria vaccines. In addition, it is also acknowledged that control and elimination of *P. vivax*, the most widespread of the *Plasmodium* sp. causing human disease, would be accelerated by the development of a *P. vivax* vaccine. Whilst this species has not been considered in this review, it is important to note that recent developments of novel tools to study *P. vivax* liver-stage biology, such as infection and generation of *P. vivax* dormant liver-stage forms both in humanized mice (Mikolajczak et al., 2015) and *in vitro* (Dembele et al., 2014), will be of great use in identifying *P. vivax* liver-stage targets in the future.

## Conflict of Interest Statement

Adrian V. S. Hill is a named investigator on US 12/595 574 and UK PCT/GB2008/01262 novel adenovirus patent applications covering malaria vectored vaccines and immunization regimens; Rhea J. Longley, Alexandra J. Spencer, and Adrian V. S. Hill are named investigators on filed patent PCT/GB2014/053077 for novel malaria antigens.
